# Postnatal and non-invasive prenatal detection of β-thalassemia mutations based on Taqman genotyping assays

**DOI:** 10.1371/journal.pone.0172756

**Published:** 2017-02-24

**Authors:** Giulia Breveglieri, Anna Travan, Elisabetta D’Aversa, Lucia Carmela Cosenza, Patrizia Pellegatti, Giovanni Guerra, Roberto Gambari, Monica Borgatti

**Affiliations:** 1 Department of Life Sciences and Biotechnology, Biochemistry and Molecular Biology Section, University of Ferrara, Ferrara, Italy; 2 Biotechnology Center, University of Ferrara, Ferrara, Italy; 3 Operative Unit of Laboratory Analysis, University Hospital S. Anna, Ferrara, Italy; University of Naples Federico II, ITALY

## Abstract

The β-thalassemias are genetic disorder caused by more than 200 mutations in the β-globin gene, resulting in a total (β^0^) or partial (β^+^) deficit of the globin chain synthesis. The most frequent Mediterranean mutations for β-thalassemia are: β^0^39, β^+^IVSI-110, β^+^IVSI-6 and β^0^IVSI-1. Several molecular techniques for the detection of point mutations have been developed based on the amplification of the DNA target by polymerase chain reaction (PCR), but they could be labor-intensive and technically demanding. On the contrary, TaqMan^®^ genotyping assays are a simple, sensitive and versatile method suitable for the single nucleotide polymorphism (SNP) genotyping affecting the human β-globin gene. Four TaqMan^®^ genotyping assays for the most common β-thalassemia mutations present in the Mediterranean area were designed and validated for the genotype characterization of genomic DNA extracted from 94 subjects comprising 25 healthy donors, 33 healthy carriers and 36 β-thalassemia patients. In addition, 15 specimens at late gestation (21–39 gestational weeks) and 11 at early gestation (5–18 gestational weeks) were collected from pregnant women, and circulating cell-free fetal DNAs were extracted and analyzed with these four genotyping assays. We developed four simple, inexpensive and versatile genotyping assays for the postnatal and prenatal identification of the thalassemia mutations β^0^39, β^+^IVSI-110, β^+^IVSI-6, β^0^IVSI-1. These genotyping assays are able to detect paternally inherited point mutations in the fetus and could be efficiently employed for non-invasive prenatal diagnosis of β-globin gene mutations, starting from the 9^th^ gestational week.

## Introduction

The β-thalassemias are genetic disorder characterized by absence (β^0^) or reduced (β^+^) synthesis of β-globin chains [1–4] associated with a corresponding excess of the complementary α-globins. The outcome of this unbalanced globin production is the destruction of erythroid precursors in bone marrow and at extramedullary sites (ineffective erythropoiesis) by apoptosis and short survival of red blood cells (RBCs) in the peripheral blood [4–6]. The disease is associated with morbidity and mortality due to severe chronic anemia or treatment-related complications. The maintenance therapeutic strategies consist of blood transfusion and pharmacological chelation, while the only current definitive therapy is bone marrow transplantation is a definitive therapy [7,8].

More than 200 mutations have been identified in β-thalassemia patients within the β-globin gene located on chromosome 11, resulting in a total (β^0^) or partial (β^+^) deficit of the β-globin chain synthesis and a subsequent quantitative absence or reduction of the production of adult HbA hemoglobin [6,7]. The most frequent Mediterranean mutations for β-thalassemia are β^0^39 (C→T), β^+^IVSI-110 (G→A), β^+^IVSI-6 (T→C) and β^0^IVSI-1 (G→A) [6,7].

The early identification of the pathogenic alteration could be relevant for verifying whether personalized therapies are available or will be expected to be available, such as the antisense approach targeting splicing mutations or the read-through approach for nonsense mutations [9–12]. This would allow responsible decision about possible termination of the pregnancy.

Also, the discrimination of these genetic hereditary mutations could be useful for carrier parents in view of a targeted prenatal diagnosis.

Several molecular techniques for the detection of point mutations have been developed based on the preventive amplification of the DNA target by polymerase chain reaction (PCR), such as restriction fragment length polymorphisms (RFLP-PCR), amplification refractory mutation system-PCR (ARMS-PCR), high resolution melting-PCR (HRM-PCR), nested-PCR, DNA sequencing [13]. However, some of these techniques could be labor-intensive and technically demanding. On the contrary, TaqMan^®^ genotyping assays are a simple, sensitive and versatile method suitable for the single nucleotide polymorphism (SNP) genotyping affecting the human β-globin gene [14].

Moreover non-invasive prenatal diagnosis (NIPD) has become increasingly important, because, although it retains only a predictive value, it allows investigating fetal health status without risks for the fetus or the mother and for early therapeutic approach [15]. NIPD is based on the discovery of circulating cell-free fetal DNA within maternal plasma [16] and, even though a huge number of studies has been performed to develop experimental approaches for the detection of fetal abnormalities at an early gestational age, such as nested PCR, digital PCR, next generation sequencing, MALDI-TOF mass spectrometry, pyrophosphorolysis-activated polymerization [17–21], no commercial assays for NIPD are available to recognize single point mutations, as currently the non-invasive screening tests offered to pregnant women can detect only aneuploidies, fetal sex, small deletions or insertions [22–24], despite the fact that promising studies are available, such as that reported by Yenilmez et al. [25] and demonstrated to be able to identify non invasively β-thalassemia mutation by HRM analysis.

The aim of the present investigation is to design, optimize and test specific genotyping assays in order to detect the four single point mutations in β-globin gene causing β-thalassemia in the Mediterranean area (β^0^39, β^+^IVSI-110, β^+^IVSI-6 or β^0^IVSI-1) in genomic DNA extracted from β-thalassemia patients before routine transfusion.

Finally, in order to perform NIPD of fetal thalassemia point mutations inherited from the father, the four SNP genotyping assays were applied to circulating DNA isolated from pregnant women plasma at different gestational ages having normal genotype and carrier partner for single point mutations in β-globin gene. In fact the identification of paternally-inherited sequences has been one of the first issues addressed after the discovery of circulating cell-free fetal DNA in maternal plasma, because its detection is easy and without maternal DNA contamination [24,26].

## Materials and methods

### Samples collection

Blood samples (25 mL) collected from healthy subjects (25), healthy carriers (33) and β-thalassemia patients (36) before routinely blood test or transfusion respectively at the Thalassemic Day Hospital (DHT) of the University Hospital “Sant’Anna” (Cona, Ferrara) and the Hospital “Santa Maria della Misericordia” (Rovigo) after approval by ethic committees “Comitato Etico della Provincia di Ferrara”, Italy and “Comitato Etico per la sperimentazione clinica delle province di Verona e Rovigo”, Italy. In this case participants were between 21 and 42 years old, 105 total approaches, 10% drop-out rate. About 20–25 ml of peripheral blood, were collected in Vacutainer LH treated tubes (Becton Dickinson), containing EDTA as anticoagulant. For prenatal experiments, whole peripheral blood (20 mL) from pregnant women (age range: 29–43 years; total maternal approaches: 27; drop-out rate: 7%) were collected, using test tubes containing EDTA as anticoagulant, in collaboration with the Laboratory of Chemical and Clinical Analysis and Microbiology, University Hospital “Sant’Anna” (Cona, Ferrara) and after approval by ethic committee “Comitato Etico della Provincia di Ferrara”, Italy. Peripheral blood or buccal swabs were obtained by future fathers carrying thalassemia point mutations and accompanying the future mother for her routinely blood work interested to the project. The infant buccal swabs were collected from mother that received the swab procedure at the time of her blood sample and sent us the obtained infant salivary swab after delivery. In all these cases the informed and written consents were obtained from the mother or father at the time of taking maternal blood draw previously approval by ethic committee “Comitato Etico della Provincia di Ferrara”, Italy. Document participant consents were recorded by physician performed the blood sampling at the University Hospital “Sant’Anna” (Cona, Ferrara) and the Hospital “Santa Maria della Misericordia” (Rovigo). Baseline demographic characteristics of the study populations were not recorded. All the experiments were conducted in agreement with the Declaration of Helsinki.

### Genomic DNA extraction

Genomic DNA was extracted from 0.5 ml of blood or two buccal swabs using the QIAamp^®^ DNA Blood Mini Kit (Qiagen, Hilden, Germany), according to manufacturer’s instructions. For the blood, 50 μl of Qiagen Protease and 500 μl of buffer AL were added to 500 μl of blood; after an incubation at 56°C for 10 minutes and brief centrifugation, 500 μl of 96% ethanol were mixed to the sample. On the contrary, in the case of buccal swabs, the cotton swab was cut with a scalpel, and then 400 μl of PBS (Phosphate Buffered Saline), 20 μl of Qiagen Protease and 400 μl of buffer AL were added; the mixture was incubated at 56°C for 1 hour and after a brief centrifugation, 400 μl of 96% ethanol were mixed to the sample. At this point, the same procedure was applied following the manufacturer’s protocols. Finally the DNA was checked by agarose-gel electrophoresis, spectrophotometrically quantified, and stored at -20°C.

### Circulating cell-free DNA extraction

Plasma was prepared within 3 hours from 20 mL blood collection, according to the protocol described in literature [27]. Briefly, after mixing tubes in a rotator for 5–10 minutes, samples were centrifuged at 1200 x g for 10 minutes at 4°C without brake. Plasma was then carefully collected and centrifuged again at 2400 x g for 20 minutes at 4°C in order to completely remove platelets and precipitates. The resulting supernatant (6–8 mL) was collected and stored at -80°C into single-use 2 mL aliquots. DNA was extracted from 2 ml of maternal plasma, not thawed more than once, by using the QIAamp^®^ DSP Virus Spin Kit (Qiagen), according to the manufacturer’s instructions. The availability of several 2 mL aliquots of serum allowed comparison of different DNA extraction kits and will allow in the future validation of different mutation detection protocols.

### Polymerase Chain Reaction (PCR)

Each reaction was prepared in a final volume of 50 μl, containing ExTaq Buffer (Takara, Otsu, Shiga, Japan), dNTPs, 150 ng PCR primers, 1.25 U/reaction ExTaq DNA polymerase (Takara). After a first denaturation step at 94°C for 2 minutes, amplification cycles included denaturation at 94°C for 30 seconds, annealing at a 1–2 degrees lower than the melting temperature of primers for 30 seconds, and elongation at 72°C for a time proportional to the product length, taking into account that the enzyme inserts, on average, 1000 bp/minute; at the end, the reactions were maintained at 72°C for 10 minutes to complete the process of elongation. In each PCR reaction aimed to DNA sequencing, 100 ng or 30 ng of human genomic DNA obtained from blood or buccal swabs, respectively, were amplified by using three pairs of primers (Table 1), in order to include the whole β-globin gene. 0.8 mM dNTPs were employed, and 35 amplification cycles were performed.

**Table 1:** List of primers employed to amplify and sequence the β-globin gene.

Sequence	Tm	PCR length (bp)
5'-GTGCCAGAAGAGCCAAGGACAGG-3'	72.1°C	641
5'-AGTTCTCAGGATCCACGTGCA-3'	67.1°C	
5'-GCCTGGCTCACCTGGACA-3'	67.9°C	954
5'-GTTGCCCAGGAGCTGTGG-3'	67.2°C	
5'-ACAATCCAGCTACCATTCTGCTTT-3'	65.7°C	436
5'-CACTGACCTCCCACATTCCCTTTT-3'	69.9°C	

The nucleotide sequence and the melting temperature (Tm) are reported for each oligonucleotide. For every pair of primers the length of the respective PCR product (bp, base pairs) is indicated as well.

PCR products were analyzed by agarose-gel electrophoresis, purified using MicroCLEAN (Microzone Limited, Haywards Heath, West Sussex, UK) before the DNA sequencing. On the contrary, in each PCR reaction aimed to pre-amplification for prenatal diagnosis, 1 μl of circulating DNA extracted from maternal plasma was amplified by using a specific pair of primers, according to the mutation under investigation (Table 2) and in order to increase fetal DNA template quantity in subsequent genotyping assays.

**Table 2:** List of primers employed to amplify circulating DNA in non-invasive prenatal diagnosis experiments.

Sequence	Tm	PCR length (bp)	Mutation
5'-TTAGGCTGCTGGTGGTCTA-3'	55.5°C	81	β^0^39
5'-CCATAACAGCATCAGGAGTGG-3'	55.4°C		
5'-AGAGAAGACTCTTGGGTTTCTGATAG-3'	55.7°C	74	β^+^IVSI-110
5'-GCAGCCTAAGGGTGGGAAA-3'	57.6°C		
5'-CAAGGTGAACGTGGATGAAGTT-3'	55.5°C	96	β^+^IVSI-6
5'-CATGCCCAGTTTCTATTGGTCTC-3'	55.7°C		
5'-CAAGGTGAACGTGGATGAAGTT-3'	55.5°C	105	β^0^IVSI-1
5'-CTGTCTCCACATGCCCAGTTT-3'	57.6°C		

The nucleotide sequence and the melting temperature (Tm) are reported for each oligonucleotide. For every pair of primers, the length of the respective PCR product (bp, base pairs) and the relative mutation are also indicated.

0.4 mM dNTPs were employed, and 30 amplification cycles were performed. Annealing temperature was 61°C for primers aimed to detect the β^0^39 mutation, and 55°C for the other pairs. 1 μl of PCR product was used as a template for each genotyping assay, without previous analysis.

### TaqMan^®^ genotyping assays

Every reaction was performed in a final volume of 15 μl containing DNA template (1 ng of genomic DNA or 1 μl of pre-amplification reactions of circulating DNA obtained from maternal plasma), the specific amplification genotyping assay and a PCR Master Mix (Thermo Fisher Scientific, Waltham, MA, USA). The nucleotide sequences of primers and probes are reported in Table 3.

**Table 3:** List of oligonucleotides used as primers or probes for genotyping experiments.

Sequence	Primer/Probe	Mutation
5'-CTTAGGCTGCTGGTGGTCTAC-3'	Forward primer	β039
5'-AGTGGACAGATCCCCAAAGGA-3'	Reverse primer	
5'-AAGAACCTCTGGGTCCAA-3'	VIC^®^ probe	
5'-CAAAGAACCTCTAGGTCCAA-3'	FAM^™^ probe	
5'-GGGTTTCTGATAGGCACTGACT-3'	Forward primer	β+IVSI-110
5'-GCAGCCTAAGGGTGGGAAA-3'	Reverse primer	
5'-CTCTGCCTATTGGTCTAT-3'	VIC^®^ probe	
5'-TCTCTGCCTATTAGTCTAT-3'	FAM^™^ probe	
5'-GTGAACGTGGATGAAGTTGGT-3'	Forward primer	β+IVSI-6
5'-CTATTGGTCTCCTTAAACCTGT-3'	Reverse primer	
5'-CTTGTAACCTTGATACCAACC-3'	VIC^®^ probe	
5'-TGTAACCTTGATGCCAACC-3'	FAM^™^ probe	
5'-GTGAACGTGGATGAAGTTGG-3'	Forward primer	β0IVSI-1
5'-CCCAGTTTCTATTGGTCTCCTTA-3'	Reverse primer	
5'-TGGGCAGGTTGGTA-3'	VIC^®^ probe	
5'-TGGGCAGATTGTAT-3'	FAM^™^ probe	

The nucleotide sequence and the relative investigated mutation are reported.

All the reactions were performed in duplicate. For each analysis, no-template controls were prepared as well. The reactions were carried out on a StepOne^™^ Real-Time PCR System (Applied Biosystems^®^—Thermo Fisher Scientific), by using the StepOne Software (Applied Biosystems^®^—Thermo Fisher Scientific). After a first 2-minute cycle at 50°C and a second 10-minute cycle at 95°C, the amplification cycles were different for each specific genotyping assay: 50 amplification cycles with denaturation at 95°C for 15 seconds, and annealing and elongation at 62°C for 1 minute for β^0^39, β^+^IVSI-110 and β^+^IVSI-6 assays; 60 amplification cycles with denaturation at 95°C for 15 seconds, and annealing and elongation at 60°C for 1 minute for β^0^IVSI-1. Since each reaction was performed in duplicate, the average Rn value was calculated for each sample. For each genotyping assay, final VIC^®^ and FAM^™^ Rn values were plotted in an allelic discrimination plot.

For analysis of genomic DNA, the end-point fluorescence produced by VIC^®^ and FAM^™^ was normalized with the ROX^™^ reference, according to the following equations: VIC^®^ Rn = VIC^®^/ROX^™^; FAM^™^ Rn = FAM^™^/ROX^™^.

### DNA sequencing

β-globin PCR products obtained using, as target, genomic DNA from healthy donors, healthy carriers, hemoglobinopathy patients, future fathers, or from babies, were sequenced according to Sanger’s method [28] with primers reported in Table 1. Sequence reactions were performed in final volume of 20 μl, containing 40–90 ng of PCR template, 3.2 pmol of sequencing primer and ultrapure water to a 12 μl volume. Then 8 μl of Terminator Ready Reaction Mix of ABI PRISM^®^ BigDye^™^ Terminator Cycle Sequencing Ready Reaction Kit (Thermo Fisher Scientific), were added, containing the four differently-labeled dideoxyribonucleotides (ddNTPs), the AmpliTaq^®^ DNA polimerase, MgCl_2_ and Tris-HCl buffer at pH 9.0. A total of 45 amplification cycles were performed, as follows: denaturation, 96°C, 10 s; annealing, 65°C, 5 s; elongation, 65°C, 3 min. The reaction products were then purified from unincorporated ddNTPs by using a 96-well MultiScreen^™^ (Merck Millipore, Billerica, MA, USA) plate: after loading on pre-hydrated wells filled with Sephadex^™^ G-50 Superfine (GE Healthcare, Little Chalfont, UK), samples were recovered by centrifugation at 900 x g for 6 minutes and dried under vacuum. Sequencing was finally performed by BMR Genomics (Padova, Italy), while the obtained sequence data were analyzed by the Sequence Scanner, version 1.0 (Applied Biosystems^®^—Thermo Fisher Scientific), software.

## Results

### Genotyping assays for the detection of β-thalassemia point mutations in genomic DNA

The first aim of this work was the development of a postnatal approach for molecular diagnosis of β-thalassemia point mutations based on TaqMan^®^ genotyping assays.

This approach was already presented in a previous study by Alwazani et al. [14]; this study was, however, not focused on NIPD.

Each custom assay contains two primers, able to recognize and amplify both the normal and the mutated sequence, and two differently labeled TaqMan^®^ probes: a probe specific for the normal sequence, labeled at the 5’ end with VIC^®^ (4,7,2’-trichloro-7’phenyl-6-carboxy-fluorescein), and a FAM^™^ (6-carboxy-fluorescein)-conjugated probe able to hybridize with the mutated sequence [29]; at the 3′ end, both the probes are coupled with the quencher NFQ, a non-fluorescent quencher acting as energy transfer acceptor [30].

We designed four genotyping assays for the detection of the most common Mediterranean mutations for β-thalassemia in genomic DNA extracted from blood or buccal swabs of 94 donors with different clinical conditions.

**Fig 1:**
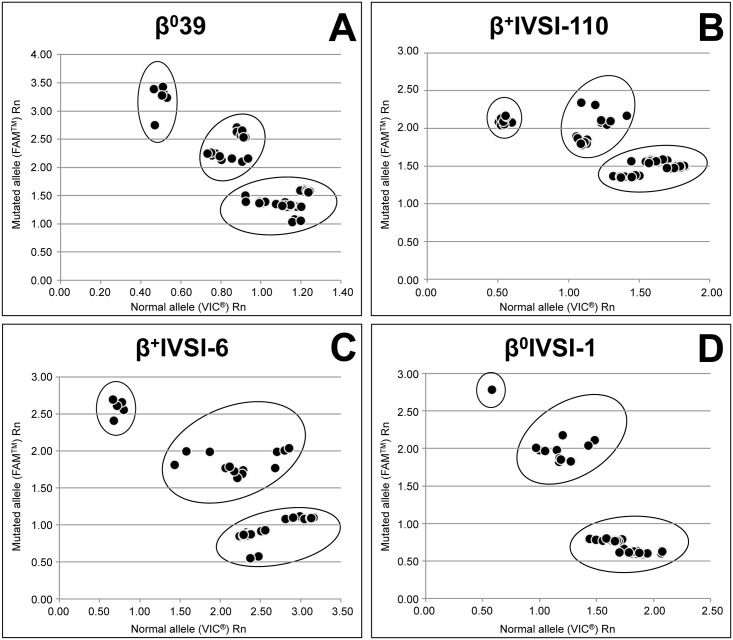
Allelic discrimination plots obtained from 94 total samples of genomic DNA. The genomic DNAs extracted from healthy donors, healthy carriers and β-thalassemia patients, were analyzed with β^0^39 (A), β^+^IVSI-110 (B), β^+^IVSI-6 (C), or β^0^IVSI-1 (D) genotyping assays. For each plot, the normalized end-point fluorescence (Rn) values generated by the VIC^®^-labeled probe for the normal allele and the FAM^™^-labeled probe for the mutated allele are reported along the x-axis and the y-axis, respectively.

We prepared 25 samples from healthy subjects, 33 samples from healthy carriers and 36 samples from β-thalassemia patients with homozygous or heterozygous genotype for the four mutations, all confirmed by DNA sequencing (Fig 1 and Table 4). Each sample was analyzed by all four single-plex genotyping assays specific for one of the β-thalassemia single point mutation.

**Table 4:** List of β-globin allele genotypes resulting by genotyping assays.

Analyzed subjects	Genotype	No. samples
Healthy donors	N/N	25
Healthy carriers	β^0^39/N	11
	β^+^IVSI-110/N	11
	β^+^IVSI-6/N	7
	β^0^IVSI-1/N	4
β-thalassemia patients	β^0^39/β^0^39	8
	β^+^IVSI-110/β^+^IVSI-110	8
	β^+^IVSI-6/β^+^IVSI-6	5
	β^0^IVSI-1/β^0^IVSI-1	1
	β^+^IVSI-6/β^+^IVSI-110	2
	β^+^IVSI-110/β^0^39	3
	β^0^IVSI-1/β^0^39	3
	β^+^IVSI-6/β^0^39	2
	β^+^IVSI-110/β^0^IVSI-1	1
	β^+^IVSI-6/β^0^IVSI-1	3

The analysis was performed for 94 total samples of genomic DNA from healthy donors, healthy carriers and β-thalassemia patients. For each genotype, the number of samples deriving from blood or buccal swabs is indicated as well.

In particular Fig 1 reports the allelic discrimination plots generated by the genotyping assays specific for β^0^39 (A), β^+^IVSI-110 (B), β^+^IVSI-6 (C), β^0^IVSI-1 (D). All the diagrams show that the samples are arranged into three major and distinct groups in the allelic discrimination plot: samples from healthy subjects (N/N), having a high value of VIC^®^ fluorescence and a low value of FAM^™^ fluorescence, are arranged in the lower right position; in contrast homozygous samples for a specific mutation (Mut/Mut), are arranged in the upper left position characterized by high FAM^™^ and low VIC^®^ fluorescence values, respectively. Finally, samples from healthy carriers (N/Mut) and double heterozygous subjects (Mut/Mut*), giving similar values of FAM^™^ and VIC^®^ fluorescences, are placed in the middle. We never observed exceptions to this discrimination results and genotype-associated patterns.

In addition, in all cases no differences were found among results obtained by DNA samples purified by blood or buccal swabs (data not shown), indicating that the latter may be considered a very interesting simple and non-invasive alternative to blood sampling as a source of genomic DNA for molecular diagnosis.

### Genotyping assays for the detection of β-thalassemia point mutations in circulating cell-free fetal DNA

**Fig 2:**
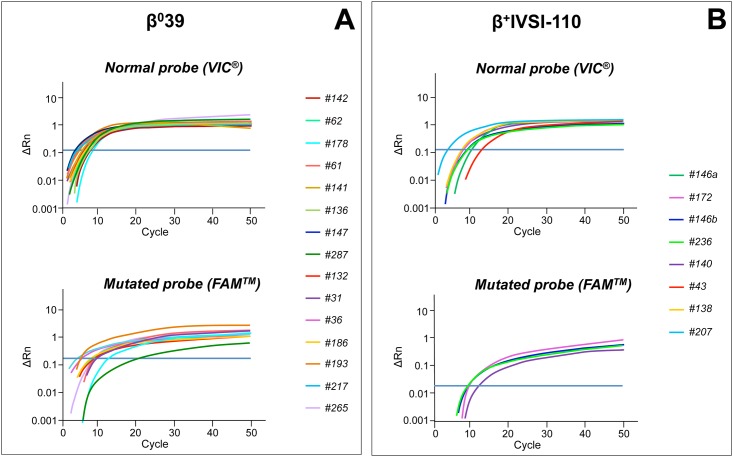
Amplification curves obtained by β^0^39 or β^+^IVSI-110 genotyping assays from circulating DNA extracted from maternal plasma. Real-time PCR with β^0^39 (A) or β^+^IVSI-110 (B) genotyping assays, containing a normal probe (VIC^®^, upper panels) and a mutated probe (FAM^™^, lower panels) were performed for circulating DNA extracted from maternal plasma where the father was a carrier of β^0^39 (A) or β^+^IVSI-110 (B) thalassemia mutation, respectively. The plots show the ΔRn values as a function of the number of amplification cycles, while the threshold line is drawn in blue. Samples are listed according to increasing gestational ages. In panel (B), #146a and #146b refer to samples collected from the same pregnant woman (number 146) at different gestational ages: 5 weeks and 10 weeks, respectively. The amplification was performed by using the StepOne^™^ Real-Time PCR System (Applied Biosystems^®^—Thermo Fisher Scientific).

**Fig 3:**
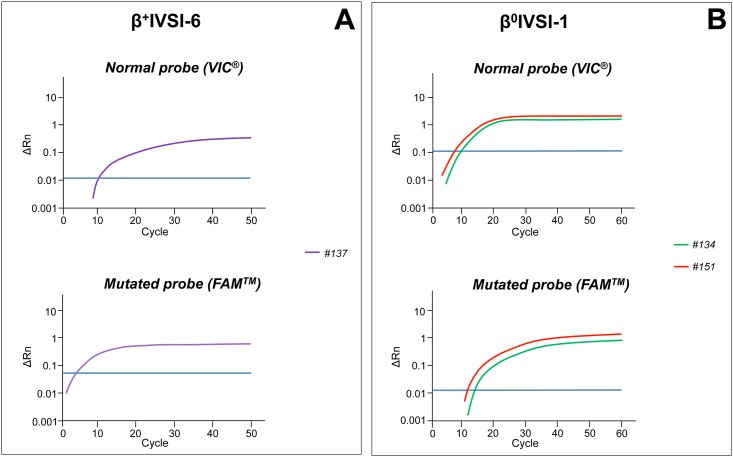
Amplification curves obtained by β^+^IVSI-6 or β^0^IVSI-1 genotyping assays from circulating DNA extracted from maternal plasma. Real-time PCR with β^+^IVSI-6 (A) or β^0^IVSI-1 (B) genotyping assays, containing a normal probe (VIC^®^, upper panels) and a mutated probe (FAM^™^, lower panels), were performed for circulating DNA extracted from maternal plasma where the father was a carrier of β^+^IVSI-6 (A) or β^0^IVSI-1 (B) thalassemia mutation, respectively. The plots show the ΔRn values as a function of the number of amplification cycles, while the threshold line is drawn in blue. Samples are listed according to increasing gestational ages. The amplification was performed by using the StepOne^™^ Real-Time PCR System (Applied Biosystems^®^—Thermo Fisher Scientific).

Twenty-six samples of circulating fetal DNA obtained from pregnant women at different gestational ages with father carrier of β-thalassemia were analyzed by TaqMan^®^ genotyping assays (Figs 2 and 3 and Table 5). In particular, we collected 15 specimens at late gestation (21–39 gestational weeks) and 11 specimens at early gestation (5–18 gestational weeks), in order to test the assay sensitivity, as the concentration of cell-free fetal circulating DNA raises in maternal circulation with increasing gestational age [31].

**Table 5:** List of samples of circulating DNA extracted from maternal plasma with father carrier for β thalassemia point mutation, analyzed by the specific genotyping assay.

**β**^**0**^**39 mutation**					
*# sample*	*Gestational age (weeks)*	*Father's genotype*	*Formulated diagnosis*	*Fetus‘ genotype*	*Diagnosis outcome*
142	5	β^0^39/N	N/N	β^0^39/N	Wrong
62	13	β^0^39/N	β^0^39/N	β^0^39/N	Confirmed
178	14	β^0^39/N	β^0^39/N	β^0^39/N	Confirmed
61	21	β^0^39/N	β^0^39/N	β^0^39/N	Confirmed
141	22	β^0^39/N	N/N	N/N	Confirmed
136	23	β^0^39/N	N/N	N/N	Confirmed
147	24	β^0^39/N	N/N	N/N	Confirmed
287	24	β^0^39/N	β^0^39/N	β^0^39/N	Confirmed
132	25	β^0^39/N	β^0^39/N	β^0^39/N	Confirmed
31	26	β^0^39/N	β^0^39/N	β^0^39/N	Confirmed
36	28	β^0^39/N	β^0^39/N	β^0^39/N	Confirmed
186	29	β^0^39/N	β^0^39/N	β^0^39/N	Confirmed
193	33	β^0^39/N	β^0^39/N	β^0^39/N	Confirmed
217	35	β^0^39/N	β^0^39/N	β^0^39/N	Confirmed
265	39	β^0^39/N	β^0^39/N	β^0^39/N	Confirmed
**β**^**+**^**IVSI-110 mutation**					
*# sample*	*Gestational age (weeks)*	*Father's genotype*	*Formulated diagnosis*	*Fetus‘ genotype*	*Diagnosis outcome*
146a	5	β^+^IVSI-110/N	N/N	β^+^IVSI-110/N	Wrong
172	9	β^+^IVSI-110/N	β^+^IVSI-110/N	β^+^IVSI-110/N	Confirmed
146b	10	β^+^IVSI-110/N	β^+^IVSI-110/N	β^+^IVSI-110/N	Confirmed
236	10	β^+^IVSI-110/N	β^+^IVSI-110/N	n.d.	n.d.
140	15	β^+^IVSI-110/N	β^+^IVSI-110/N	β^+^IVSI-110/N	Confirmed
43	18	β^+^IVSI-110/N	N/N	N/N	Confirmed
138	37	β^+^IVSI-110/N	N/N	N/N	Confirmed
207	37	β^+^IVSI-110/N	N/N	N/N	Confirmed
**β**^**+**^**IVSI-6 mutation**					
*# sample*	*Gestational age (weeks)*	*Father's genotype*	*Formulated diagnosis*	*Fetus‘ genotype*	*Diagnosis outcome*
137	11	β^+^IVSI-6/N	β^+^IVSI-6/N	n.d.	n.d.
**β**^**0**^**IVSI-1 mutation**					
*# sample*	*Gestational age (weeks)*	*Father's genotype*	*Formulated diagnosis*	*Fetus‘ genotype*	*Diagnosis outcome*
134	17	β^0^IVSI-1/N	β^0^IVSI-1/N	β^0^IVSI-1/N	Confirmed
151	28	β^0^IVSI-1/N	β^0^IVSI-1/N	β^0^IVSI-1/N	Confirmed

The father was a carrier of β^0^39, β^+^IVSI-110, β^+^IVSI-6 or β^0^IVSI-1 thalassemia mutation, analyzed with the specific β^0^39, β^+^IVSI-110, β^+^IVSI-6 or β^0^IVSI-1 genotyping assay. Samples are listed according to increasing gestational ages. #146a and #146b refer to samples collected from the pregnant woman #146 at 5 and 10 gestational weeks, respectively. The father genotype, the fetus genotype, the formulated diagnosis by genotyping assays and the diagnosis outcome are reported. n.d., not determined.

In order to identify the mutations of the β-globin gene of carrier fathers, a buccal swab or a blood sample was collected from the future father and genomic DNAs were extracted and sequenced to identify the specific genotype.

In our case, the identified mutations cover the most common Mediterranean alterations causing β-thalassemia, and also within this small group of samples, the relative frequency is the same: the majority of fathers carried the β^0^39 mutation, a lower number the β^+^IVSI-110 and only a few the β^0^IVSI-1 and β^+^IVSI-6 mutations (Table 5).

As reported in literature [22–26], the detection of fetal mutations inherited from the father should be quite feasible because they are not present in maternal DNA, that is the main component of circulating DNA extracted from maternal plasma. In the case of genotyping assays, with mutation transmitted by the father, maternal circulating DNA does not carry the mutated sequence (the mother’s genotype is N/N) and so will be amplified only by the VIC^®^-labeled probe complementary to the normal sequence. Therefore, if the fetus is N/N like the mother, only an amplification signal from the VIC^®^-labeled probe is expected; while if the fetus has inherited the father’s mutation, his circulating DNA, in addition to be amplified by the normal VIC^®^-labeled probe, will be recognized also by the FAM^™^-labeled probe carrying the mutated sequence. In conclusion, the generation of a FAM^™^ fluorescence signal, related to the presence of a mutated allele, should occur only in the case the fetus has inherited the father’s mutation.

Moreover fetal DNA represents a small portion of the total circulating DNA ranging between 3 and 6% [32], despite the fact that a percentage close to 10–20% in the last weeks of gestation has been recently reported [31,33]. To overcome this drawback and avoid false negative results, we assayed a pre-amplification approach aimed to increase the target fetal sequences before the genotyping assays. Circulating DNA sample, after pre-amplification, was analyzed using the genotyping assay specific for the relative father’s mutation and the identified fetal genotype was confirmed by sequencing the newborn genomic DNA obtained from buccal swab (Table 5).

With respect to the β^0^39 mutation, 15 samples from pregnant women at different gestational ages having β^0^39 carrier fathers were collected. The FAM^™^-labeled mutated probe allowed to identify mutated alleles for all samples, suggesting a heterozygous β^0^39/N fetal genotype, except for four samples (#142, #141, #136, #147), where no mutated curves were detectable in accordance with a N/N genotype (Fig 2A).

All the identified fetal genotypes were confirmed except for the sample #142 (Table 5). In this case, the actual baby’s genotype was β^0^39/N, but no mutated curve was obtained by genotyping assay (Fig 2A). Perhaps this depends on the too low amount of fetal DNA in the specimens collected at early stage pregnancy (5 gestational weeks).

The same problem occurred in the β^+^IVSI-110 diagnosis, where no mutation-dependent signals were produced by sample #146a at the 5^th^ gestational week, in contrast with the relative β^+^IVSI-110/N genotype obtained after postnatal baby’s DNA sequencing (Table 5). While the same sample collected at the 10^th^ gestational week (#146b) showed the correct fetus’ genotype, confirming that the circulating cell-free fetal DNA at 5 gestational weeks is really scarce for a correct detection by genotyping assay. As for the other six samples, the FAM^™^-labeled mutated probe allowed to identify mutated alleles only for samples #172, #236 and #140, suggesting heterozygous β^+^IVSI-110/N genotypes and normal genotypes for the other three samples (#43, #138, #207) (Fig 2B). All these fetus genotypes were confirmed after sequencing of the child’s genomic DNA from buccal swabs, except for sample #236 due to not obtaining of buccal swabs of father and infant (Table 5).

Unfortunately, for the β^+^IVSI-6 and β^0^IVSI-1 mutations, only one and two samples were available, respectively. For the mutation β^+^IVSI-6 the only sample #137 was analyzed. The pre-amplified circulating DNA generated detectable amplification curves with both normal and mutated probes (Fig 3A); therefore we could propose the heterozygous β^+^IVSI-6/N genotype of a β-thalassemia carrier: this diagnosis is still waiting for confirmation because the buccal swab of the child has not obtained yet (Table 5). For the β^0^IVSI-1 mutation, all mutated alleles were identified by the FAM^™^-labeled mutated probe (Fig 3B), showing for both samples heterozygous β^0^IVSI-1/N genotypes, as confirmed by sequencing the newborns’ genomic DNAs (Table 5).

## Discussion

Hemoglobin disorders are recognized among the most common inherited diseases worldwide. Approximately 7% of the world population currently exhibits symptoms of hemoglobinopathies, including β-thalassemia. The early identification of the pathogenic molecular alterations could be useful for verifying whether personalized therapies are available or will be expected to be available allowing responsible decision about possible termination of the pregnancy, as well as the active search of clinicians those personalized interventions.

Several techniques based on polymerase chain reaction (PCR) or genomic sequencing are currently used for molecular diagnosis of thalassemia, but they are labor-intensive and time-consuming in performing the assays and analyzing the results. On the contrary, rapid and accurate molecular techniques are essential for detection of thalassemia mutations in affected families.

In this context, the TaqMan^®^ genotyping assay represents a reliable, sensitive and cost-effective diagnostic method allowing the discrimination of single nucleotide polymorphisms [34,35]. Thus, the TaqMan^®^ genotyping assay is suitable for large-scale screenings and high-throughput analyses. The simplicity and reproducibility of the assay permit its use in laboratories as a rapid (it allows detection of mutations in less than 40 minutes) and inexpensive diagnostic tool for mutations diagnosis [35].

It requires two subsequent steps: a first amplification by PCR or real-time PCR, in the presence of suitable probes, followed by the detection of the end-point resulting signals. To distinguish the two different alleles, a pair of allele-specific oligonucleotide probes are used, one complementary to the normal sequence and the other specific for the mutated one, marked with different fluorescent labels.

TaqMan^®^ genotyping assays were designed for characterization of β-thalassemia mutations in the Malays and Kingdom of Saudi Arabia [14,34–36]. Also, an analogous TaqMan^®^ assay was developed and reported to be effective for detection of the Sardinian Wilson’s mutation [37]. In addition, TaqMan^®^ genotyping probes can be designed to detect also multiple nucleotide polymorphisms and insertions/deletions. For example, this approach has been suggested as a rapid and reliable assay for genotyping of different α-globin gene deletions, also applicable to mass screening for probable carriers [38].

In this work, we designed four TaqMan^®^ genotyping assays for the most common β-thalassemia mutations in the Mediterranean area (β^0^39, β^+^IVSI-110, β^+^IVSI-6, β^0^IVSI-1). The different genotypes of 36 thalassemia patients were characterized using these assays and all confirmed by DNA sequencing. In addition, the TaqMan^®^ genotyping assays for the β^0^39, β^+^IVSI-110, β^0^IVSI-1 mutations in thalassemia patients have been recently validated for the detection of the three genetic variants in patients from western region of the Kingdom of Saudi Arabia [14]. However these reports were focused on post-natal diagnostic approaches, while the results presented in this paper appears to retain considerable interest in the filed of rapid prenatal screening.

In recent years, several non-invasive approaches based on the detection of circulating cell-free fetal DNA in maternal plasma have been developed to carry out prenatal diagnosis [17–21,39], but currently, commercial non-invasive prenatal screening tests are very expensive, besides requiring technical expertise and complex equipment and infrastructure. In addition, they only allow the detection of fetal sex and the most common aneuploidies, but not point mutations causing genetic disorders [33,40–42].

In this context, genotyping assays could be an alternative and robust tool for the genetic diagnosis of single point mutations causing genetic diseases such as thalassemia.

Therefore we applied the four genotyping assays to non-invasive prenatal detection of thalassemia fetal point mutations inherited from the father, because fetal paternally-inherited sequences in maternal plasma present relative easiness of detection as they are not present in maternal contaminating DNA [22–26].

The fraction of circulating cell-free fetal DNA in maternal plasma is a critical parameter for genetic screening with non-invasive prenatal testing, especially at the earlier gestational age. Based on these considerations, we performed a pre-amplification step before the genotyping assays in order to avoid false negative results and increase target fetal sequences.

Our results indicate that the developed genotyping assays are able to detect paternally-inherited point mutations in the fetus, and could be efficiently employed for non-invasive prenatal diagnosis of β-globin gene mutations, starting from the 9^th^ gestational week. The obtained data demonstrated that genotypic discrimination at earlier gestational ages (< 5^th^ week) by genotyping assays was prevented, probably due to the very low amount of fetal circulating DNA.

Since circulating fetal DNA is detectable in maternal plasma from 4^th^-5^th^ week of gestation [43], the improvement of DNA extraction and enrichment procedures [44,45] could be proposed in order to apply genotyping assays in earlier phases of gestation.

It should be mentioned that alternative, but costly, novel technologies could be proposed, such as digital PCR. For this technology, the discrimination of possible fetal mutations is apparently not prevented by the maternal DNA background [46,47]. Accordingly, a comparison of the two approaches should be considered in future experimental planning. Moreover, it will be useful the increasing of the analyzed samples, especially at early gestation, to estimate the detection limit of the technique and the application of this approach to other thalassemia or sickle cell anemia mutations.

## Conclusions

We developed and validated four simple, inexpensive and versatile genotyping assays for the postnatal and prenatal identification of the most common Mediterranean mutations causing β-thalassemia: β^0^39, β^+^IVSI-110, β^+^IVSI-6, β^0^IVSI-1. The developed genotyping assays are able to detect paternally-inherited point mutations in the fetus, and could be efficiently employed for non-invasive prenatal diagnosis of β-globin gene mutations, starting from the 9^th^ gestational week. It should be useful the increasing of the analyzed samples, especially at early gestation, to estimate the detection limit of the technique and the application of this approach to other thalassemia or sickle cell anemia mutations.
